# Essential Oil of *Bursera morelensis* Promotes Cell Migration on Fibroblasts: In Vitro Assays

**DOI:** 10.3390/molecules28176258

**Published:** 2023-08-26

**Authors:** Judith Salas-Oropeza, Marco Aurelio Rodriguez-Monroy, Manuel Jimenez-Estrada, Armando Perez-Torres, Andres Eliu Castell-Rodriguez, Rodolfo Becerril-Millan, Katia Jarquin-Yanez, Maria Margarita Canales-Martinez

**Affiliations:** 1 Laboratorio de Farmacognosia, UBIPRO Facultad de Estudios Superiores Iztacala, UNAM, Tlalnepantla C.P. 54090, Mexico; judithsalazo@hotmail.com (J.S.-O.); rbecerrilm85@gmail.com (R.B.-M.); 2 Laboratorio de Investigación Biomédica en Productos Naturales, Carrera de Medicina Facultad de Estudios Superiores-Iztacala, UNAM, Tlalnepantla C.P. 54090, Mexico; dr.marcorodriguezmonroy@gmail.com; 3 Instituto de Química-UNAM, Circuito Exterior, Ciudad Universitaria, Ciudad de México D.F. 04510, Mexico; manuelj@unam.mx; 4 Facultad de Medicina-UNAM, Circuito Exterior, Ciudad Universitaria, Ciudad de México D.F. 04510, Mexico; armandop@unam.mx (A.P.-T.); castell@unam.mx (A.E.C.-R.); katys12@hotmail.com (K.J.-Y.)

**Keywords:** *Bursera*, essential oil, cell migration, wound healing

## Abstract

Essential oils (EOs) are complex mixtures of volatile natural compounds. We have extensively studied the EO of *Bursera morelensis*, which demonstrates antibacterial, antifungal, anti-inflammatory, and wound-healing activities. The objective of this work was to determine the effect of this EO on fibroblast migration in a three-dimensional in vitro model. For the three-dimensional in vitro model, a series of fibrin hydrogel scaffolds (FSs) were built in which fibroblasts were cultured and subsequently stimulated with fibroblast growth factor (FGF) or EO. The results demonstrated that these FSs are appropriate for fibroblast culture, since no decrease in cell viability or changes in cell proliferation were found. The results also showed that this EO promotes cell migration four hours after stimulation, and the formation of cell projections (filopodia) outside the SF was observed. From these results, we confirmed that part of the mechanism of action of the essential oil of *B. morelensis* during the healing process is the stimulation of fibroblast migration to the wound site.

## 1. Introduction

Plants synthesize low-molecular weight organic compounds called secondary metabolites, which include large groups of compounds, such as phenols, terpenes, and those containing nitrogen. These metabolites are often lineage-specific and help plants interact with biotic and abiotic environments [1,2]. This is the case for aromatic plants, which synthesize and accumulate complex mixtures of volatile natural compounds, which consist mainly of terpenoid hydrocarbons, oxygenated terpenes, sesquiterpenes, aromatic and aliphatic terpenoids, and other constituents called essential oils (EOs) [3,4]. The use of essential oils has been predominantly linked to perfumes, cosmetics, and food flavorings due to their high aroma. In addition, continuous research has demonstrated the immense potential of essential oils and their constituent chemical species in the management, protection, and cure of several human diseases [4].

Our research group has extensively studied the EO of *Bursera morelensis*, a tree in the *Burseraceae* family traditionally used to treat wounds, and the results of several of our works have confirmed the different biomedical activities of both the EO and some of its constituent compounds, demonstrating antibacterial, antifungal, anti-inflammatory, and wound-healing activities [5,6,7,8]. In terms of wound healing, we proposed that the mechanism of action of this EO is the promotion of fibroblast migration to the wound site, making these fibroblasts active in the production of collagen and promoting collagen remodeling [8].

Wound healing includes all of the processes necessary for the recovery of a wound. The study and understanding of wound healing has been divided into three sequential phases, each of which has its own period of time, as well as the involvement of particular tissues and cell lineages [9,10,11,12]. The first is the inflammatory phase. In this stage, hemorrhage stops with the formation of a clot composed of platelets and fibrin; likewise, defense mechanisms (neutrophils, macrophages, and lymphocytes) are activated [13,14,15]. The healing process continues in the proliferative phase, in which epidermal, endothelial, and fibroblasts migrate and proliferate at the injury site [15,16], which generates initial granulation tissue [17].

During granulation tissue formation, fibroblasts migrate, proliferate, and synthesize large amounts of collagen and other parts of the extracellular matrix to fill the dermal defect in a process known as fibroplasia [18]. Subsequently, new blood vessels are formed (angiogenesis), and capillary sprouting associated with fibroblasts and macrophages replaces the fibrin matrix with granulation tissue, which forms a new substrate for cell migration. Vascular endothelial growth factor A (VEGFA) and fibroblast growth factor 2 (FGF2) are the most important positive regulators of angiogenesis [19].

Fibroblasts are the key cells responsible for constructing granulation tissue to fill in the wound gap [17]. In response to various signaling molecules that are released from tissue-resident macrophages, platelets, keratinocytes, and endothelial cells, fibroblasts proliferate, migrate, and become pro-fibrotic, depositing extracellular matrix (ECM) [20]. Prior to laying ECM proteins by fibroblasts, they obliterate provisional matrix by secreting matrix metalloproteinases, which are substituted by granulation tissue rich in collagen, fibronectin, glycoproteins, and proteoglycans [19]. Finally, the remodeling stage occurs; during this phase, as the wound heals, capillary growth stops, fibroblast and macrophage density is reduced by apoptosis, and blood flow and metabolic activity decrease. This remodeling process of scar tissue lasts approximately a year or even longer; by the end of the stage, a fully mature scar with a reduced number of cells and blood vessels and high tensile strength has formed [17,19,20].

Therefore, the objective of this work was to determine the effect of the EO of *B. morelensis* on fibroblast migration in a three-dimensional in vitro model.

## 2. Results

### 2.1. Cell Viability

**Figure 1:**
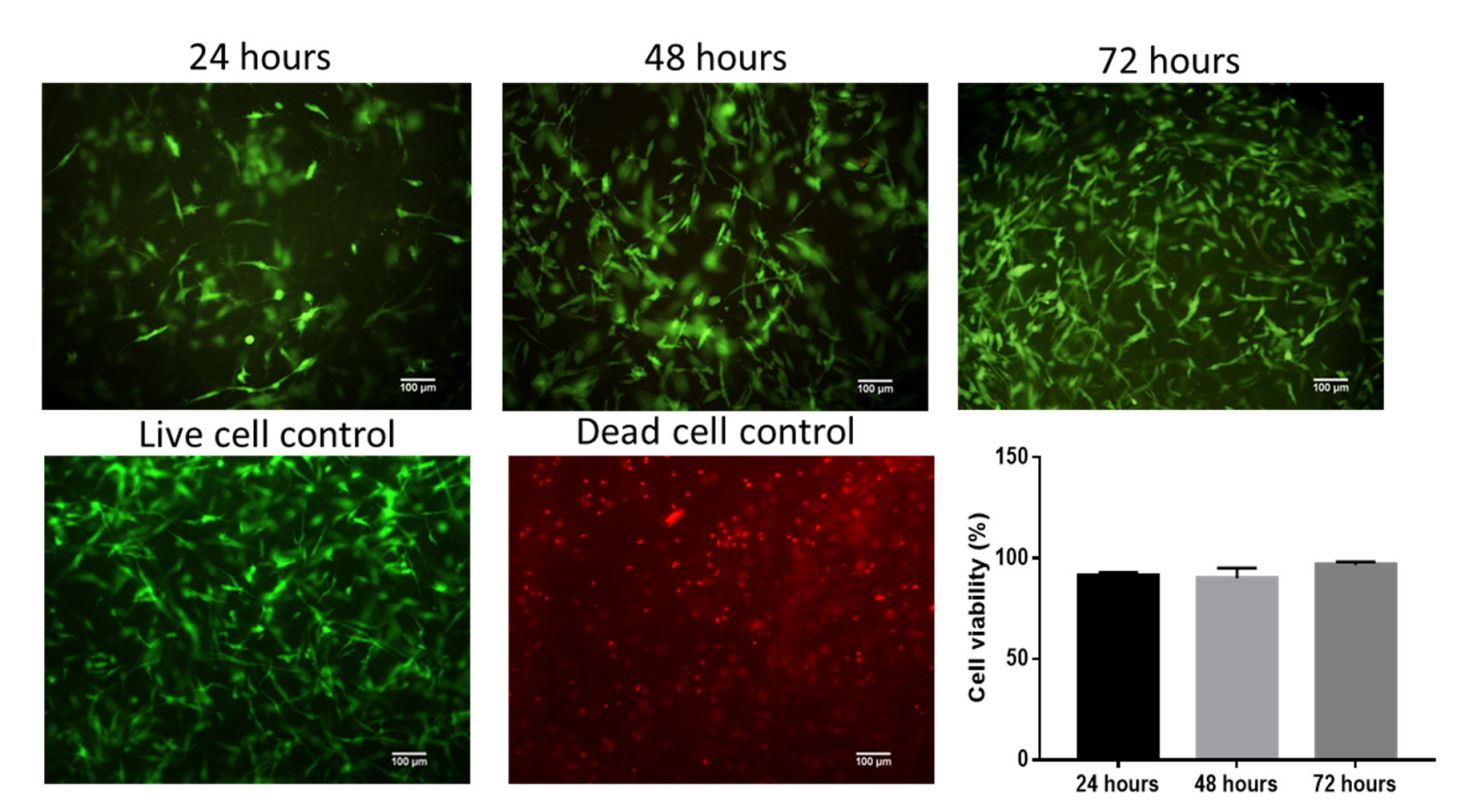
Viability test. Live/dead staining of fibroblasts cultured on FS for 24 h, 48 h, and 72 h after stimulation with the EO of *B. morelensis* (0.01 g/mL).

To verify cell viability on the fibrin scaffold (FS), live/dead staining was performed after applying the EO (0.01 mg/mL) every 24 h for three days. The results showed that the application of EO did not affect cell viability, since at three time points (24, 48, and 72 h), few red nuclei (dead cells) were observed (Figure 1). These data demonstrate that the FSs are conducive to the cultivation of fibroblasts, corroborating previous results on the effect of EO on cell viability [8,21].

### 2.2. Cell Proliferation

**Figure 2:**
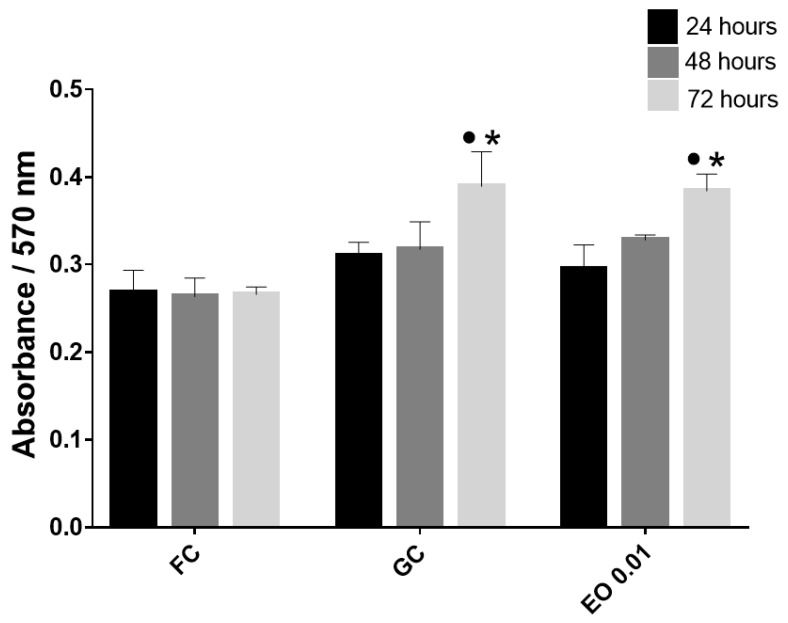
Cell proliferation assay. Resazurin absorbance indicates the proliferation of fibroblasts. Fibroblast culture conditions: FC = supplemented DMEM and FS without cells; GC = growth control (supplemented DMEM and FS with cells); and EO 0.01: 0.01 g/mL essential oil (supplemented DMEM, FS with cells and essential oil). • Significant differences with respect to FC. * Significant differences with respect to 24 h. (*p* < 0.05).

Once the viability of the fibroblast cultures on the FS was estimated, cell proliferation was evaluated by a PrestoBlue assay, where the absorbance is directly proportional to cell proliferation. The absorbance was found to increase with time (Figure 2).

**Figure 3:**
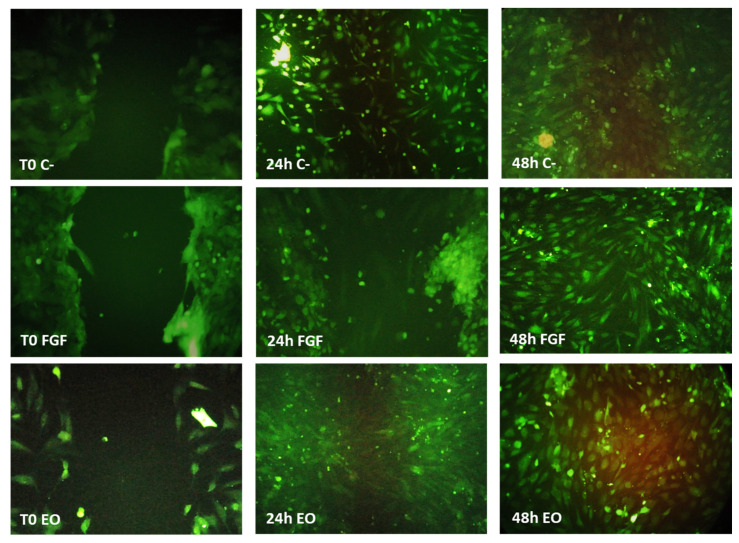
Migration assay in monolayer cultures of fibroblasts stained with carboxyfluorescein succinimidyl ester (CFSE). A wound was simulated in the center of the monolayer, and the following stimuli were immediately applied: M: supplemented DMEM; FGF: growth factor fibroblasts; and essential oil (EO): 0.01 mg/mL. The photographs correspond to time 0 (immediately after wound generation), 24 h after wound generation, and 48 h after wound generation.

To monitor cell migration, fibroblasts were stained with carboxyfluorescein succinimidyl ester (CFSE), which confers live, active cells with green fluorescence. Once the cells were stained, they were cultured in a plate until they reached confluence. Then, a wound was simulated and monitored for 48 h. Figure 3 clearly shows that after 24 h, there was migration to the wound area in all the stimulus groups. In the plate with EO, it was also observed that migration was very localized, as clusters of cells at the ends of the wound were not introduced into the empty space.

To verify that EO promotes fibroblast migration, a FS migration assay was carried out using a double scaffold within polymerized scaffolds in six-well plates. A pair of wells was embedded with a separation distance of 1 mm; one of these scaffolds had fluorescently stained cells, and one of three stimuli was added to the other: DMEM, FGF, or 0.01 mg/mL EO.

Cell migration is a highly integrated multistep process that is initiated by protrusion of the cell membrane. Protrusive structures formed by migrating and invading cells are termed filopodia, lamellipodia, or invadopodia/podosomes, depending on their morphological, structural, and functional characteristics. Lamellipodia are flat and sheet-like membrane protrusions formed at the leading edge of migrating cells. It is generally believed that lamellipodia have a major role in driving cell migration by attaching to the substrate and generating force to pull the cell body forward. Filopodia are thin, finger-like projections consisting of bundled, crosslinked actin filaments. Filopodia are also observed at the migrating front of cells [22].

**Figure 4:**
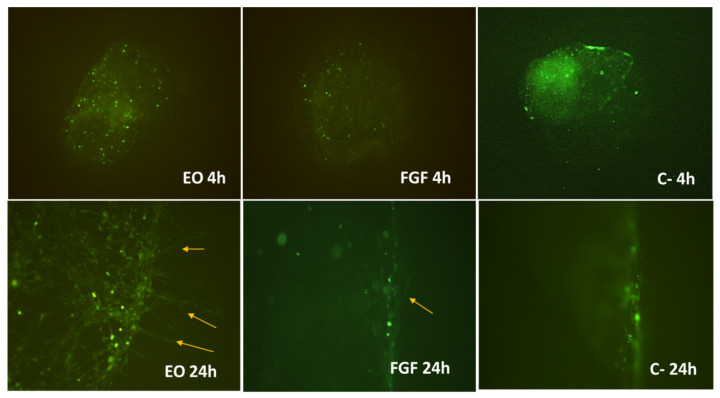
Cell culture migration on fibrin hydrogel scaffolds with fibroblasts stained with carboxyfluorescein succinimidyl ester (CFSE) 4 h (4 h) and 24 h (24 h) after scaffold assembly. EO: essential oil; FGF: fibroblast growth factor; and C-: negative control fibroblasts grown in stimulus-free growth medium. Yellow arrows indicate the projections of the fibroblasts outside the fibrin scaffold in the essential oil and fibroblast growth factor samples.

In the images in Figure 4, after 24 h, the fibroblasts began to leave the first scaffold and extend in the direction where the stimulus was applied. This phenomenon was particularly evident in the EO group. The shape and dimensions of the fibroblast projections observed in the scaffolds treated with EO and FGF suggest that they are filopodia.

## 3. Discussion

Cell migration is a fundamental biological function of living cells for the establishment and maintenance of the proper organization of multicellular tissues. Cell migration is associated with the continuous assembly and disassembly of focal adhesions (FAs), which mediate signal transduction or adhesion to the ECM and are regulated by specific signaling pathways. Cell migration is a complex process that can be influenced by a variety of factors, which can be divided into two broad categories: chemical factors and mechanical factors [23,24,25]. During the wound repair process, endothelial cells and fibroblasts migrate to the site of injury and accumulate granulation tissues, depositing collagen and other ECM. During the final stages of repair, fibroblasts remodel collagen by producing matrix metalloproteinases (MMPs) over several months [26].

Due to the importance of cell migration during wound repair, in this work, we constructed a three-dimensional model of migration in vitro, in which fibroblasts isolated from the skin of healthy donors [8] were cultured on fibrin scaffolds. The cell viability test (live/dead) showed that the FSs are suitable for culturing fibroblasts. Additionally, the results showed that at low concentrations (0.01 mg/mL), EO does not affect cell viability (Figure 1), which corroborates the cytotoxicity results found in previous works [8,21]. Fibrin is a fibrillar protein formed during blood coagulation. It is primarily involved in hemostasis but also contributes to wound healing by forming a temporary matrix surrounding the injury. Due to its high biocompatibility, fibrin has been used as a vehicle and injectable biomaterial for cell transplantation to facilitate neural regeneration, as well as in the construction of natural scaffolds in tissue engineering [27].

Similar to the cell viability results, the results obtained from the cell proliferation test showed that FS allowed cell proliferation, which was triggered at 72 h, as was observed in previous studies with monolayer fibroblast cultures [8,21]. Likewise, EO did not influence cell proliferation (Figure 2).

To monitor the migration of fibroblasts, these cells were stained with CFSE, and a preliminary migration test was performed. The results of this preliminary test can be seen in Figure 3. At 24 h, the area of the simulated wound was filled with fibroblasts. We ruled out that this result was due to proliferation, since as mentioned above, proliferation notably occurs up to 72 h (Figure 1). Due to the results from the preliminary monolayer assay, the FS assay was monitored for a maximum period of 24 h. The positive control used in this experiment was FGF (10 ng/mL). Three-dimensional (3D) scaffolds are commonly used for drug delivery, investigations of cell behavior, and material studies in the field of tissue engineering [28].

Fibrin possesses remarkable advantages over other biomaterials, and it is nature’s nanoscaffold following tissue injury to initiate hemostasis and provide a temporary structure that facilitates cellular activities and the deposition of a new extracellular matrix. Fibrin gel precursors, fibrinogen and thrombin, can be derived from a patient’s own blood, which enables the fabrication of completely autologous and inexpensive scaffolds. Moreover, owing to its multiple interaction sites for cells and other proteins, fibrin acts as a bioactive matrix and is suitable for cell and biomolecule delivery systems [29].

Four hours after application of the stimuli, fibrin scaffolds containing cells stained with CFSE were observed. At 24 h, cellular projections that moved toward the stimuli were observed. It should be noted that the cells in the negative control (unstimulated) did not show projections; therefore, we can assume that the stimulus is what promotes cell migration, and this effect was greater and more evident in fibroblasts stimulated with EO (0.01 mg/mL) (Figure 4).

The presence of filopodia is crucial. They clearly demonstrate the stimulation of migration, because filopodia act as sensors for the cell environment, investigating adjacent cells as well as the soluble cues and extracellular matrix composition and mechanical properties. These finger-like protrusions [30] are involved in both physiological and pathological cell migration and invasion in a 3D environment [31].

Cells are inherently equipped with internal compasses that respond to physical and chemical gradients within their immediate microenvironment; however, the exact molecular mechanisms that orchestrate these processes are not well understood and are an ongoing field of research [32,33,34].

Fibroblasts are typically found in connective tissue, where they synthesize collagens, glycosaminoglycans, and other important glycoproteins of the ECM, including fibronectin. These cells have been the objects of extensive in vitro study because of the ease of culturing them. Fibroblasts cultured on glass have a spread or spindle-shaped morphology, often characterized by several extending processes. In cell culture, fibroblasts move slowly, with an average speed of less than 1 μm/min, and they often change direction [23].

Growth factors are a class of polypeptides that regulate cell migration, proliferation, differentiation, and extracellular matrix synthesis, among which bFGF was reported to be a potent mitogen that can stimulate migration in various cell types [35,36,37].

In our previous studies using in vitro and in vivo assays, we proposed the potential mechanisms by which *B. morelensis* EO promotes wound healing through cell migration. However, in vitro wound healing assays cannot mimic the complexity of the conditions that occur during the wound-healing process in vivo. Therefore, in this assay, cell cultures on fibrin scaffolds were used in such a way that the cell migration process occurred in a three-dimensional environment. The results obtained from this in vitro assay corroborate previous results and confirm that one of the mechanisms of action of this EO is the stimulation of cell migration. Skin wound repair experiments using *Drosophila* as a model showed that almost all of the contractile force generated by adult wound connective tissues is delivered by a band of fibroblasts lying within 1–2 mm of the epidermal wound margin, as cutting and removal of the central wound granulation tissue did not alter the rate of wound healing [38].

Several studies have used different EOs to verify their potential as healing agents; however, an effect on cell migration has been reported in just a few of them. For example, in a study carried out on wounds treated with lavender EO, significant elevations in fibroblast growth factor 2 (FGF-2) and endothelial growth factor (EGF) were reported [39]. As its name suggests, an important function of FGF-2 is to induce fibroblast proliferation, whereas EGF is a signaling molecule responsible for wound contraction and epithelialization through the stimulation of fibroblast and epithelial cell migration, which is currently being investigated as a new topical therapy for chronic wounds [40]. On the other hand, nanostructures based on natural lipids (olive oil, coconut butter, or sesame oil) loaded with EO from rosemary or eucalyptus were tested in an in vitro migration/proliferation model and showed a clear effect on the migration of all samples [41]. To date, extensive studies have attempted to reveal the molecular mechanisms that regulate cell migration; however, they have remained largely unclear [22,42].

Fibroblasts are attracted to the wound to synthesize granulation tissue. This granulation tissue is composed of procollagen, elastin, proteoglycans, and hyaluronic acid (HA) and allows for the ingrowth of new blood vessels that provide nutrition and oxygen to the growing tissue and allow leukocytes to enter the wound site [43].

Myofibroblasts start to appear in the granulation tissue around the middle phase of wound healing. This matches the strong induction of contractile properties, so that cells align parallel to the mechanical tension that builds up in the granulation tissue. Myofibroblasts appear to differentiate themselves from fibroblasts by acquiring the smooth muscle cell actin isoform a-smooth muscle action. It seems that one of the two most important stimuli by which fibroblasts become myofibroblasts is mechanical tension [19,43,44].

Several studies have analyzed the effect of different essential oils and some terpene compounds on cell migration with various models and several cell types, finding that some of them favor cell migration [45,46]. In contrast, several investigations have found that cell migration is inhibited in a dependent manner by the dose of essential oil (from various species) or terpene compound [47,48,49,50]. The comparison of our results and those of other investigations leads us to assume that the inhibition or stimulation of cell migration of essential oils or terpene compounds is dose-dependent, so the probable relationship is that a higher concentration has a less positive effect on cell migration.

The fibroblast migration results, both in FSs and monolayers, demonstrate that EO promotes fibroblast migration. Cell migration is a key process in wound healing. Notably, cell migration is required for many biological processes, including embryonic morphogenesis, immune surveillance, and tissue repair and regeneration [22].

Identification of compound-induced molecular mechanisms remains a difficult and time-consuming process, which is challenging when working with natural products. However, the creation of works such as the one we are presenting here, with future prospects for methodological refinement and innovative techniques, including the analysis of the individual terpenic compounds, will contribute to overcoming this challenge in understanding the mechanisms of natural products.

## 4. Materials and Methods

### 4.1. Essential Oil

The EO used in this study is the same as that used in the previously reported wound-healing study, which was obtained using the hydrodistillation method. The chemical composition consists of terpene compounds that were identified by gas chromatography coupled with mass spectrometry [8]. Table 1 shows the chemical composition of the essential oil used in this work, as well as the 2D chemical structure of the terpenic compounds that form it (for those that have been reported).

### 4.2. Isolation of Fibroblasts

Fibroblasts were isolated from human skin donated with written informed consent. The skin was taken from healthy voluntary donors using a cylindrical scalpel in 5 mm biopsies under septic and antiseptic conditions. The skin obtained was immediately deposited in Hank’s solution with an antibiotic. In a laminar flow hood, the skin samples were cut into smaller fragments, and each fragment was grown in Dulbecco’s modified Eagle’s low-glucose medium (DMEM-LG) supplemented with 10% fetal bovine serum (FBS) and antibiotics (100 U/mL penicillin, 100 mg/mL streptomycin, and 100 mg/mL gentamicin), all from Gibco BRL (Rockville, MD, USA), and incubated at 37 °C with 5% CO_2_. The culture medium was replaced every two days. After two weeks of culture, the explants (skin fragments) were removed. The fibroblasts were cultured to approximately 80% confluence, and the cells were separated with 0.05%/0.02% trypsin/EDTA and reseeded to generate a sufficient number of cells for the subsequent tests [8].

### 4.3. Fibrin Hydrogel Scaffold (FS)

Samples of human plasma from healthy donors were provided by the blood bank of the General Hospital of Iguala, Guerrero, Mexico, from the Institute for Social Security and Services for State Workers (ISSSTE). Plasma was obtained by primary fractioning of total blood. Fibrin hydrogels were prepared from the nonpurified plasma of individual donors. A fibrin suspension of 200 μL was prepared with 136 μL of plasma, 24 μL of NaCl 0.9%, 40 μL of CaCl2 1%, and the following complements: 24 wells with 2500 cells, 24 wells with EO 0.01 mg/mL, 24 wells with FGF 10 ng/mL and 24 wells without complement and then incubated at 37 °C for 30 min to induce polymerization. Subsequently, DMEM-LG-supplemented medium was added [51].

### 4.4. Cell Viability and Proliferation

Cell viability was analyzed with fibroblasts cultured on fibrin scaffolds (FSs) through calcein and ethidium homodimer staining (LIVE/DEAD kit, Thermo Fisher Scientific, Waltham, MA, USA) according to the manufacturer’s instructions. For the assays, 5000 cells/cm^2^ were seeded in the FSs before polymerization in 96-well plates, cultured for 24 h with supplemented DMEM, and stimulated with 0.01 mg/mL EO diluted in cosmetic-grade mineral oil (MO) (Kamecare, Mexico). A dead control sample was obtained by treating cells with ethanol for 30 min before staining. Cells cultured with supplemented DMEM without EO were another control. Panoramic images (200×) were taken using a Nikon Eclipse 80i microscope (Nikon, Shinagawa, Tokyo, Japan) with NIS-Elements F4 (Nikon) software (version Ver 4.00.06, Tokyo, Japan). The total numbers of live and dead cells were counted manually. The viability ratio was calculated according to the following equation:

$$viability\;ratio=\frac{live\;cells}{live\;cells+dead\;cells}$$

For the cell proliferation assay, the cells were incubated with PrestoBlue^®^ reagent (Thermo Fisher Scientific) for 1 h, and then the supernatants were placed in 96-well plates. The absorbance of the contents of each well was measured at a wavelength of 570 nm using a spectrophotometric plate reader (Thermo Multi Skan Ascent Type 354) [8,21]. Each experiment was conducted three times.

### 4.5. Preliminary Test of Cell Migration in a Monolayer (2D)

Fibroblasts stained with carboxyfluorescein succinimidyl ester (CFSE) (Quah and Parish, 2010) were cultured in a six-well plate. Once the monolayer was confluent, the following stimuli were applied to the supplemented DMEM: essential oil (0.01 mg/mL), fibroblast growth factor (FGF) as the positive control (10 ng/mL), and medium as the negative control without stimulation. In each of these cultures, the cell monolayer was scraped in a straight line to create a ‘‘scratch’’ with a p200 pipet tip. The response of the cells was monitored by microscopic observation for 48 h by photographing at 4, 24, and 48 h [52] with a Nikon Eclipse 80i epifluorescence microscope (Nikon, Shinagawa, Tokyo, Japan) and NIS-Elements F4 software (Nikon). Panoramic images were taken using a Nikon Eclipse 80i microscope (200×) (Nikon, Shinagawa, Tokyo, Japan) with NIS-Elements F4 (Nikon) software (version Ver4.00.06, Tokyo, Japan).

### 4.6. Fibrin Hydrogel (FS) Scaffold Migration Test (3D)

Nine FSs were constructed in a 96-well plate with 5 × 103 cells stained with carboxyfluorescein succinimidyl ester (CFSE). To another nine scaffolds in which fibroblasts were not seeded, the stimuli were added as follows: three scaffolds were treated with 10 ng/mL FGF, three with 0.01 mg/mL EO, and three without any stimulus. Once all the FSs had polymerized, they were removed from the well and deposited in six-well plates (in these plates, the diameter of the well is larger) as follows. A scaffold with cells was placed in front of a scaffold with stimuli separated 1 mm apart. Once this was completed, the well was filled with a new solution for FSs and allowed to polymerize to wrap the first two scaffolds [51]. The response of the cells was monitored by observations taken at 4 and 24 h with a Nikon Eclipse 80i epifluorescence microscope (Nikon, Shinagawa, Tokyo, Japan) and NIS-Elements F4 software (Nikon). Panoramic images were taken using a Nikon Eclipse 80i microscope (200×) (Nikon, Shinagawa, Tokyo, Japan) with NIS-Elements F4 (Nikon) software (version Ver4.00.06, Tokyo, Japan).

### 4.7. Statistical Analysis

The results are expressed as the means ± standard errors of the means. All the results were subjected to the D’Agostino–Pearson test for normality, obtaining *p* values < 0.05. Once the normality of the data was verified, the results were analyzed by employing one-way analysis of variance (ANOVA) with a Tukey–Kramer multiple comparison post hoc test (*p* < 0.01) using GraphPad Prism version 8.4.0 (455).

## 5. Conclusions

The results of this study show that the mechanism of action of the essential oil of *B. morelensis* during the healing process is the stimulation of fibroblast migration to the wound site, which promotes wound contraction, collagen deposition, and subsequent remodeling.

## Figures and Tables

**Table 1:** Chemical composition of the essential oil and 2D chemical structure of the terpenic compounds.

Compound	Abundance (%)	Chemical Structure
α-Phellandrene	0.80	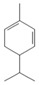
α-Pinene	8.37	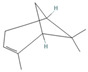
Camphene	0.13	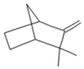
Sabinene	3.54	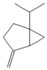
β-Myrcene *	3.60	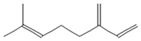
β-Phellandrene *	35.25	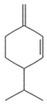
α-Terpinene	0.16	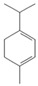
*p*-Cymene	2.10	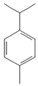
*p*-Menthane *	38.41	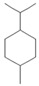
γ-Terpinene	0.18	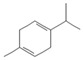
Terpinolene	0.30	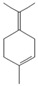
Terpinen-4-ol	0.14	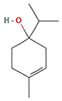
*p*-Menth-1(7)-en-2-one	0.34	NF
β-Caryophyllene	5.19	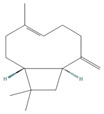
α-Caryophyllene	0.28	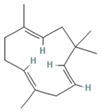
Germacrene D	0.44	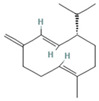
Caryophyllene oxide	0.26	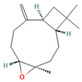
β-Eudesmol	0.14	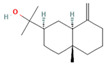

*: The dentification of these compounds is partial because the SI (SI: similarity index or match between the library and mass spectra obtained) is less than 90%.

## Data Availability

Data is contained within the article.
